# Identification of an endoplasmic reticulum stress-related prognostic risk model with excellent prognostic and clinical value in oral squamous cell carcinoma

**DOI:** 10.18632/aging.204983

**Published:** 2023-08-25

**Authors:** Mingyang Cheng, Xin Fan, Mu He, Xianglin Dai, Xiaoli Liu, Jinming Hong, Laiyu Zhang, Lan Liao

**Affiliations:** 1 The Affiliated Stomatological Hospital of Nanchang University, Nanchang, Jiangxi, China; 2 The Key Laboratory of Oral Biomedicine, Nanchang, Jiangxi, China; 3 Jiangxi Clinical Research Center for Oral Diseases, Nanchang, Jiangxi, China; 4 Clinical Medical Research Center Affiliated Hospital of Jinggangshan University, Medical Department of Jinggangshan University, Ji'An, Jiangxi, China; 5 The Stomatology College of Nanchang University, Nanchang, Jiangxi, China

**Keywords:** endoplasmic reticulum stress, prognostic risk model, oral squamous cell carcinoma, Identification, prognostic and clinical value

## Abstract

Background: Recently, endoplasmic reticulum stress related gene (ERS) markers have performed very well in predicting the prognosis of tumor patients.

Methods: The differentially expressed genes in Oral squamous cell carcinoma (OSCC) were obtained from TCGA and GTEx database. Three prognosis-related and differentially expressed ERSs were screened out by Least Absolute Selection and Shrinkage Operator (Lasso) regression to construct a prognostic risk model. Receiver Operating Characteristic Curve (ROC), riskplots and survival curves were used to verify the model’s accuracy in predicting prognosis. Multi-omics analysis of immune infiltration, gene mutation, and stem cell characteristics were performed to explore the possible mechanism of OSCC. Finally, we discussed the model’s clinical application value from the perspective of drug sensitivity.

Results: Three genes used in the model (IBSP, RDM1, RBP4) were identified as prognostic risk factors. Bioinformatics analysis, tissue and cell experiments have fully verified the abnormal expression of these three genes in OSCC. Multiple validation methods and internal and external datasets confirmed the model’s excellent performance in predicting and discriminating prognosis. Cox regression analysis identified risk score as an independent predictor of prognosis. Multi-omics analysis found strong correlations between risk scores and immune cells, cell stemness index, and tumor mutational burden (TMB). It was also observed that the risk score was closely related to the half maximal inhibitory concentration of docetaxel, gefitinib and erlotinib. The excellent performance of the nomogram has been verified by various means.

Conclusion: A prognostic model with high clinical application value was constructed. Immune cells, cellular stemness, and TMB may be involved in the progression of OSCC.

## INTRODUCTION

Oral squamous cell carcinoma (OSCC) is one of the cancers with the worst prognosis among head and neck cancers [1]. Because most cancers are advanced and have extensive metastases, their prognosis remains poor despite new diagnostics and treatments [2]. Although some progress has been made in surgery, radiotherapy, and chemotherapy of OSCC in recent years, the 5-year survival rate of OSCC is still only about 50% [3]. The standard treatment for OSCC is active resection of the primary tumor followed by adjuvant chemotherapy [4, 5]. Due to the biological characteristics of OSCC, such as engraftment and metastasis, the recurrence rate of patients is very high, and most patients are resistant to chemotherapy [6, 7].

Endoplasmic reticulum stress (ER stress) is characterized by the accumulation of misfolded and unfolded proteins in the endoplasmic reticulum cavity [8–11]. Unfolded protein reaction (UPR) play a key process in ER stress [12, 13]. UPR is crucial in regulating cell adaptation to ER stress by increasing ER content, improving ER protein folding ability and reducing misfolded protein [14, 15].

Cancer’s high metabolic demand, hypoxia, nutritional deficiency, and acidosis often lead to ER stress [16]. The intensity and duration of ER stress determine the fate of tumors [17]. Many studies have found the role of ER stress in cancer development [16]. ER stress can promote tumor proliferation and increase tumor cell invasiveness in hepatocellular carcinoma and bladder cancer [18, 19]. Some studies also found that ER stress has a positive effect on tumor treatment [20]. In renal cell carcinoma, some ER stress pharmacological modulators can change the ER stress response from pro-survival to pro-apoptosis, although this study still has defects [21]. In addition, studies also have shown that ER stress is closely related to tumor immunotherapy and tumor-infiltrating immune cells [22]. Studies have found that ER stress has multiple functions in OSCC, which can affect the proliferation and invasion of OSCC by blocking ER stress pathway, but also can inhibit the growth and reduce the survival rate of OSCC by inducing ER stress [23, 24]. Taken together, ER stress plays a crucial role in tumorigenesis and therapy, providing a new option for cancer prognosis prediction.

ER stress plays a vital role in many kinds of cancer. In addition, an increasing number of studies have confirmed the significant predictive value of ER stress related genes. Therefore, due to the limitations of various therapeutic approaches, coupled with the aggressiveness and frequent metastatic recurrence of OSCC, it is necessary to identify the abnormally expressed ER stress-related genes associated with prognosis in OSCC and construct a predictive model that can effectively predict the prognosis and treatment effectiveness of OSCC.

## RESULTS

### Identification of DE-ERSs and construction of PPI network

**Figure 1:**
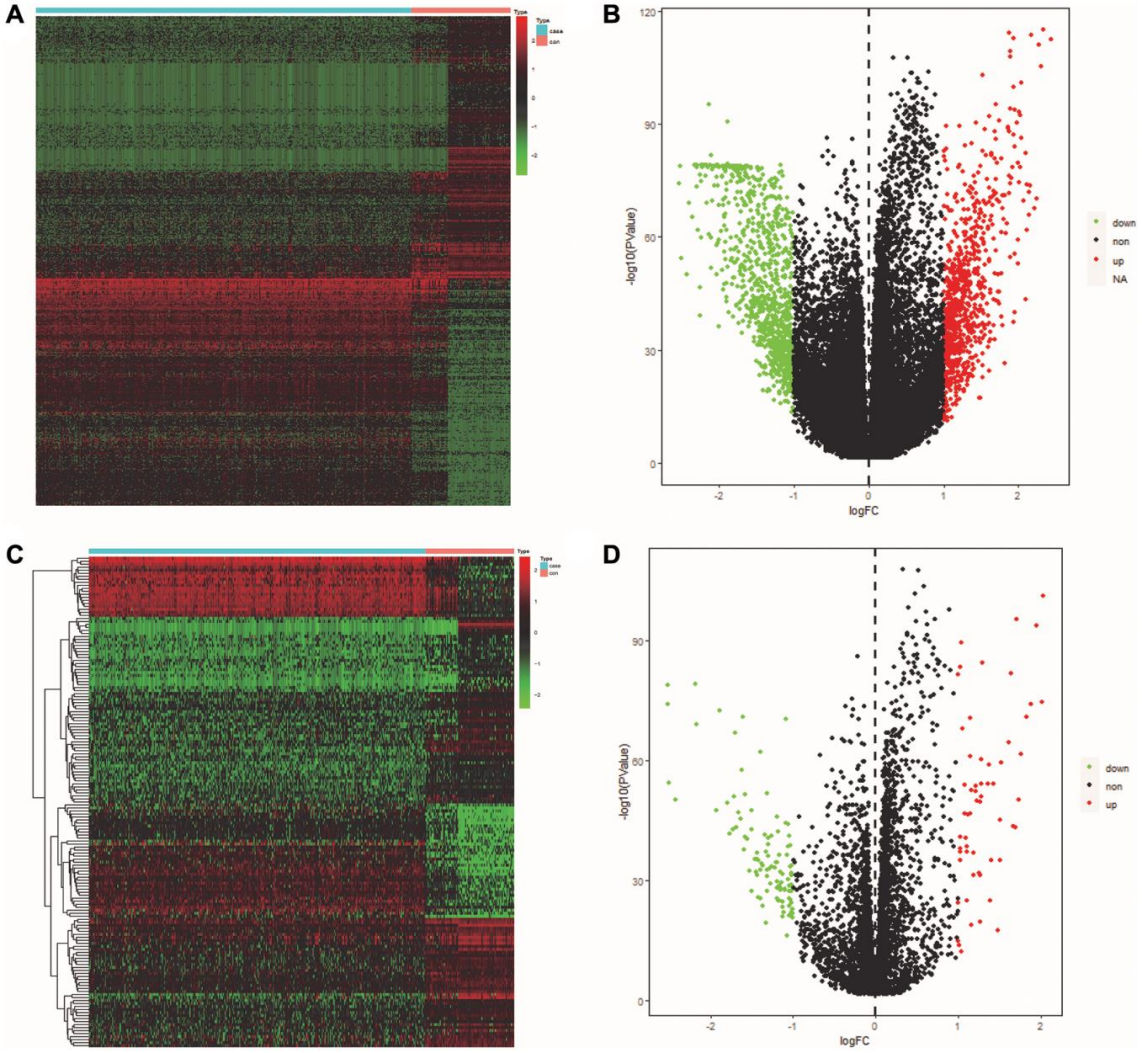
**Identification of DEGs and DE-ERSs.** (**A**) Heat map of DEGs based on TCGA OSCC data. (**B**) Volcano map of DEGs based on TCGA OSCC data. (**C**) Heat map of DE-ERSs based on TCGA and GeneCards OSCC data. (**D**) Volcano map of DE-ERSs based on TCGA and GeneCards OSCC data.

**Figure 2:**
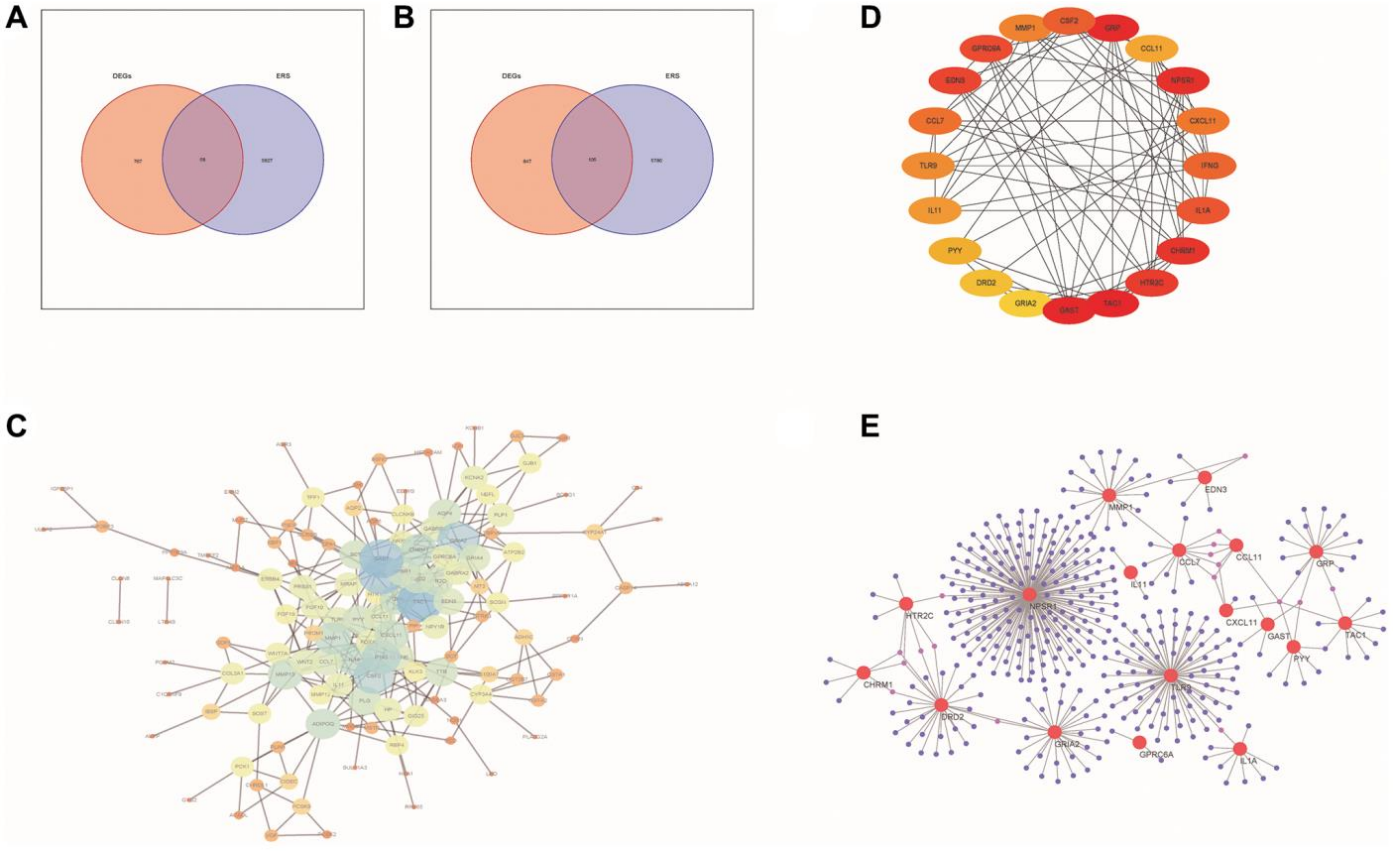
**PPI network analysis of DE-ERSs.** (**A**, **B**) Venn diagram of DE-ERSs obtained by intersecting DEGs and ERSs. (**C**) The PPI network of DE-ERSs. (**D**) Maximum correlation analysis on the top 20 hub genes by CytoHubba plug-in. (**E**) Network Diagram of top 20 hub genes visualized by NetworkAnalyzer tool.

The volcano maps and clustered heatmaps in Figure 1A, 1B showed 1,777 DEGs obtained by differential analysis, including 825 up-regulated and 925 down-regulated DEGs, respectively. We found 58 common up-regulated DE-ERSs and 105 common down-regulated DE-ERSs in the Venn diagram generated by the intersection of DEGs and ERSs in Figure 2A, 2B. They are further visualized in Figure 1C, 1D.

As shown in Figure 2C, our study generated a PPI network for DE-ERSs based on the STRING database. Not only the top 20 genes (CSF2, GRP, CCL11, NPSR1, CXCL11, IFNG, IL1A, CHRM1, HTR2C, TAC1, GAST, GRIA2, DRD2, PYY, IL11, TLR9, CCL7, EDN3, GPRC6A and MMP1) as a hub gene (Figure 2D), and their interaction relationships were also visualized by the NetworkAnalyzer tool (Figure 2E).

### Gene function annotation of DE-ERSs

**Figure 3:**
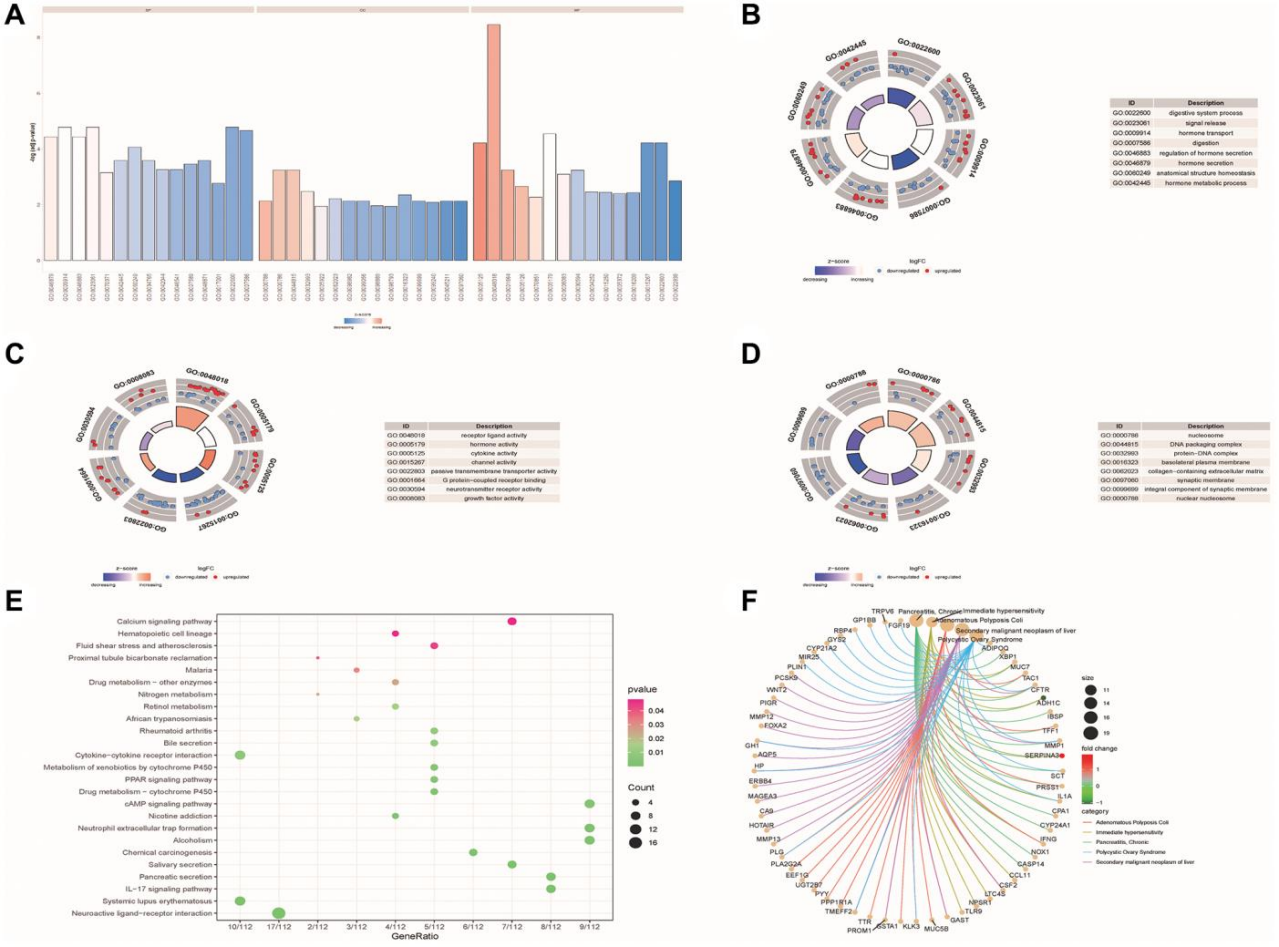
**Functional enrichment analysis of DE-ERSs.** (**A**–**D**) The GO function enrichment analysis results of DE-ERSs including BP, CC and MF. (**A**) When z-score was defined as the abscissa, -log (*p*.adjust) was defined as the ordinate. The first, second, and third parts represent BPs, CCs, and MFs. (**B**–**D**) The results of BP, CC and MF, respectively. The color of the node gene represented the level of expression in the tumor tissue. Blue indicated down-regulation of the expression value, while red indicated up-regulation of the expression value. The middle quadrilateral represented the effect of the gene on the enriched GO terms. Dark colors showed inhibition, while light colors indicated activation. (**E**) The results of KEGG pathway enrichment analysis. The node size and color indicated the number of genes enriched in the pathway and -log10 *P*-value. (**F**) The first five results of DO enrichment analysis of DE-ERSs.

To explore the role of DE-ERSs, we employed GO functional enrichment analysis to enrich the biological processes that DE-ERSs might participate in (Figure 3A).

The analysis results showed that DE-ERSs were significantly enriched in biological processes (BP) such as digestive system process, signal release, hormone transport, digestion, regulation of hormone secretion, hormone secretion, anatomical structure homeostasis and hormone metabolic process (Figure 3B). DE-ERSs were also significantly associated with cellular components (CC) such as nucleosome, DNA packaging complex, protein-DNA complex, basolateral plasma membrane, collagen-containing extracellular matrix, synaptic membrane, integral component of synaptic membrane and nuclear nucleosome (Figure 3C). In addition, many molecular functions (MF) such as receptor ligand activity, hormone activity, cytokine activity, channel activity, passive transmembrane transporter activity, G protein-coupled receptor binding, neurotransmitter receptor activity and growth factor activity was enriched (Figure 3D). DE-ERSs may also be involved in many pathways, including neuroactive ligand-receptor interaction, systemic lupus erythematosus, IL-17 signaling pathway, pancreatic secretion, Salivary secretion, chemical carcinogenesis, alcoholism, neutrophil extracellular trap formation, nicotine addiction, cAMP signaling pathway (Figure 3E). To further explore the impact of DE-ERSs on disease, we also performed a DO analysis of DE-ERSs. The results showed that DE-ERSs were significantly enriched in pancreatitis, chronic, immediate hypersensitivity, adenomatous colonic polyps, secondary liver malignancies, polycystic ovary syndrome, and other diseases (Figure 3F).

### GSEA enrichment analysis of DE-ERSs

**Figure 4:**
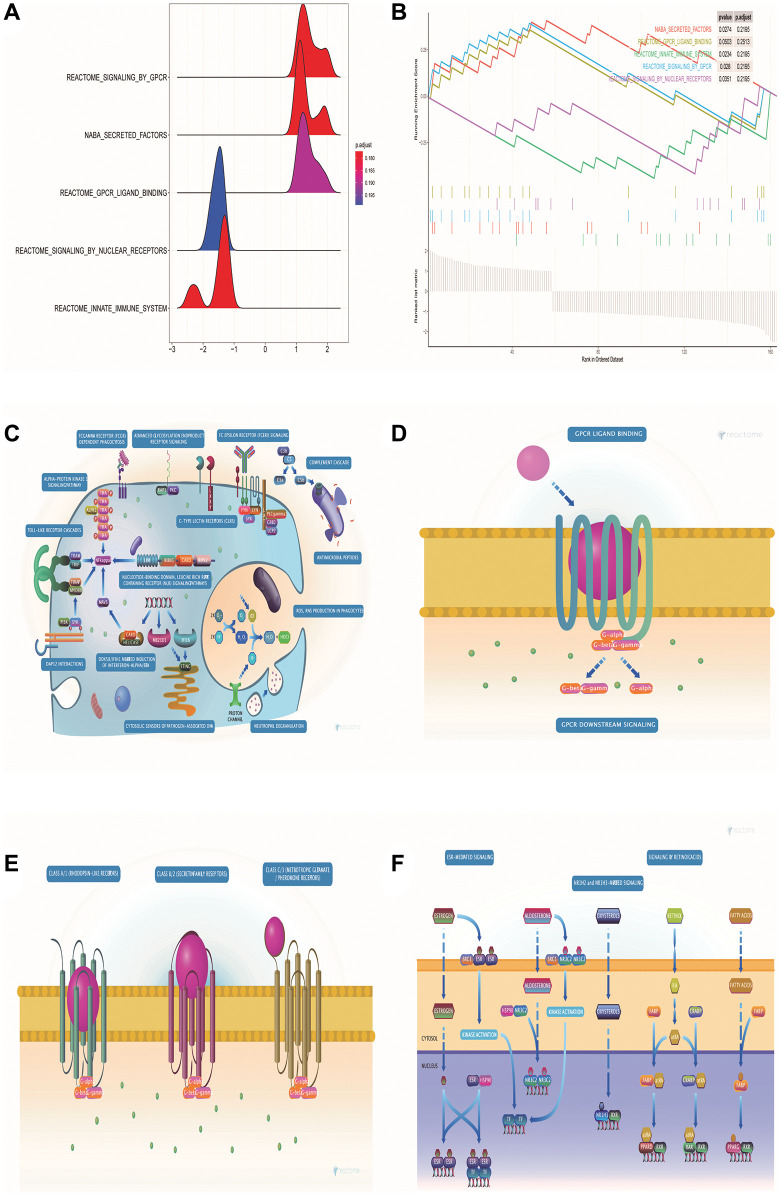
**GSEA pathway enrichment analysis of DE-ERSs.** (**A**) The mountain map shows the enrichment results of the GSEA pathway. (**B**) The top five enrichment results in the GSEA pathway enrichment analysis. (**C**–**F**) Biological process diagrams of the four pathways that GSEA pathway enrichment analysis obtained.

GSEA pathway enrichment analysis showed that DE-ERSs were significantly enriched in G protein-coupled receptor (GPCR), innate immune system, GPCR ligand binding, nuclear receptor signaling, and signaling in other pathways (Figure 4A, 4B). Figure 4C–4F showed biological processes of the innate immune system, GPCR signaling, GPCR ligand binding, and nuclear receptor signaling, respectively. These figures were downloaded from the REACTOME database (http://reactome.org/).

### Identification of 110 prognosis-related DE-ERSs

**Figure 5:**
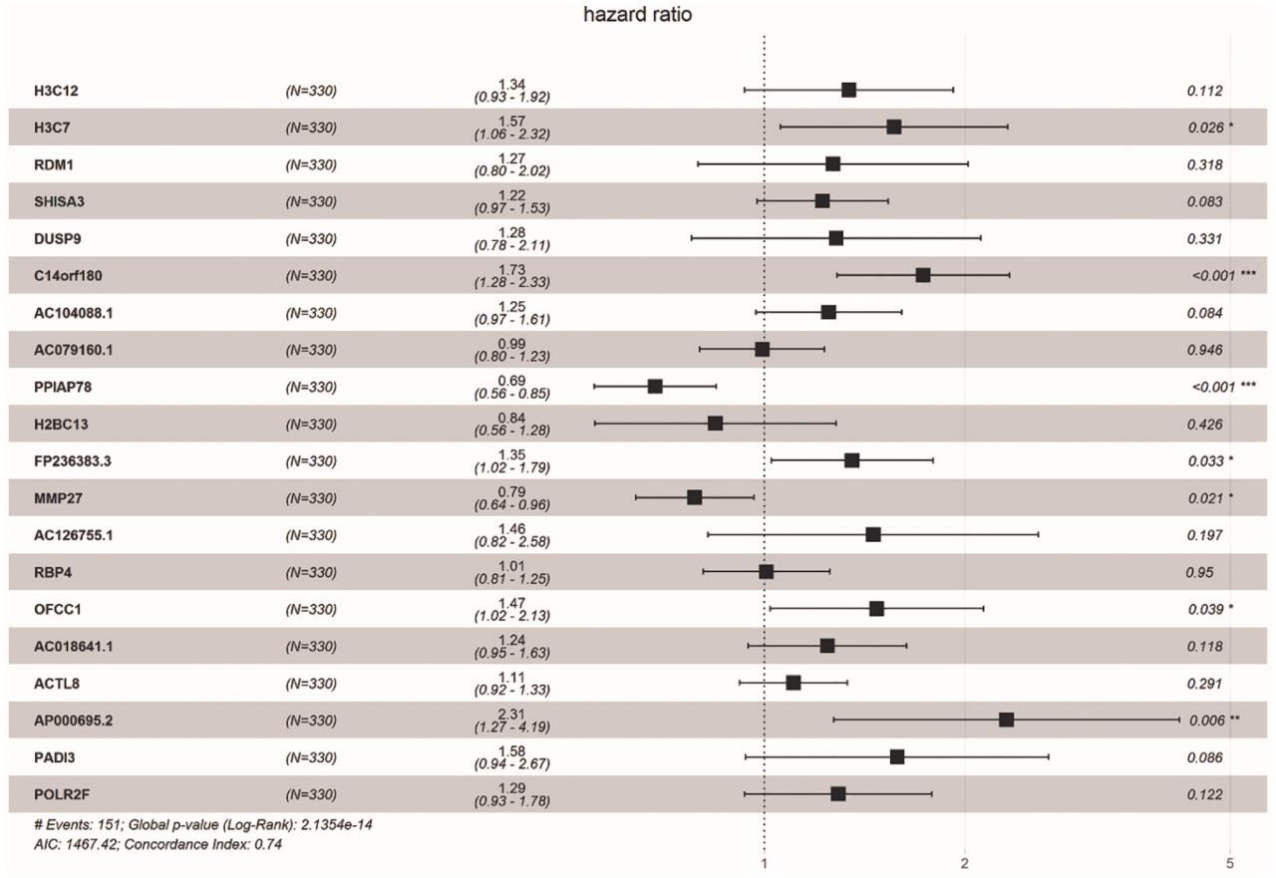
**Forest plot showing the top 20 prognosi- related DE-ERSs obtained by univariate regression analysis.** Protective genes and risk genes were located on the left and right sides of the vertical dotted line, respectively.

We obtained 18 common DE-ERSs for subsequent analysis in 110 prognoses related DE-ERSs and ER stress-related genes. Figure 5 and Table 1 showed the univariate Cox regression results for the top 20 DE-ERSs.

**Table 1:** Univariate Cox regression analysis of top 20 DEGs.

**Gene**	**Hazard_Ratio**	**CI**	***P*-value**
H3C12	1.656	1.25–2.193	0.000
H3C7	1.638	1.213–2.21	0.001
RDM1	2.026	1.335–3.074	0.001
SHISA3	1.369	1.129–1.66	0.001
DUSP9	2.136	1.326–3.441	0.002
C14orf180	1.497	1.158–1.935	0.002
AC104088.1	1.383	1.114–1.718	0.003
AC079160.1	1.304	1.088–1.563	0.004
PPIAP78	0.765	0.639–0.917	0.004
H2BC13	1.641	1.173–2.296	0.004
FP236383.3	1.496	1.141–1.964	0.004
MMP27	0.772	0.647–0.921	0.004
AC126755.1	2.138	1.276–3.582	0.004
RBP4	1.312	1.093–1.575	0.004
OFCC1	1.507	1.12–2.027	0.007
AC018641.1	1.398	1.098–1.779	0.007
ACTL8	1.261	1.064–1.494	0.007
AP000695.2	2.073	1.215–3.537	0.007
PADI3	1.94	1.199–3.139	0.007
POLR2F	1.468	1.108–1.945	0.007

### Construction and verification of prognostic risk model

After running Least Absolute Selection and Shrinkage Operator (Lasso) regression and multiple Cox regression, we constructed a prognostic risk model using the 3 prognosis-related DE-ERSs (Supplementary Figure 1A, 1B, Table 2). Finally, we applied the coefficients obtained by the lasso regression algorithm to the following risk scoring equation: risk score = IBSP × 0.428 + RDM1 × 0.962 + RBP4 × 0.305.

**Table 2:** Multivariate Cox regression analysis of endoplasmic reticulum stress related genes in oral squamous cell carcinoma.

**Gene**	**Coefficient**	**Hazard_Ratio**	**CI**	***P*-value**
IBSP	0.428	1.535	1.017–1.276	0.041
RDM1	0.962	2.617	1.023–2.315	0.009
RBP4	0.305	1.357	1.800–5.367	0.034

**Figure 6:**
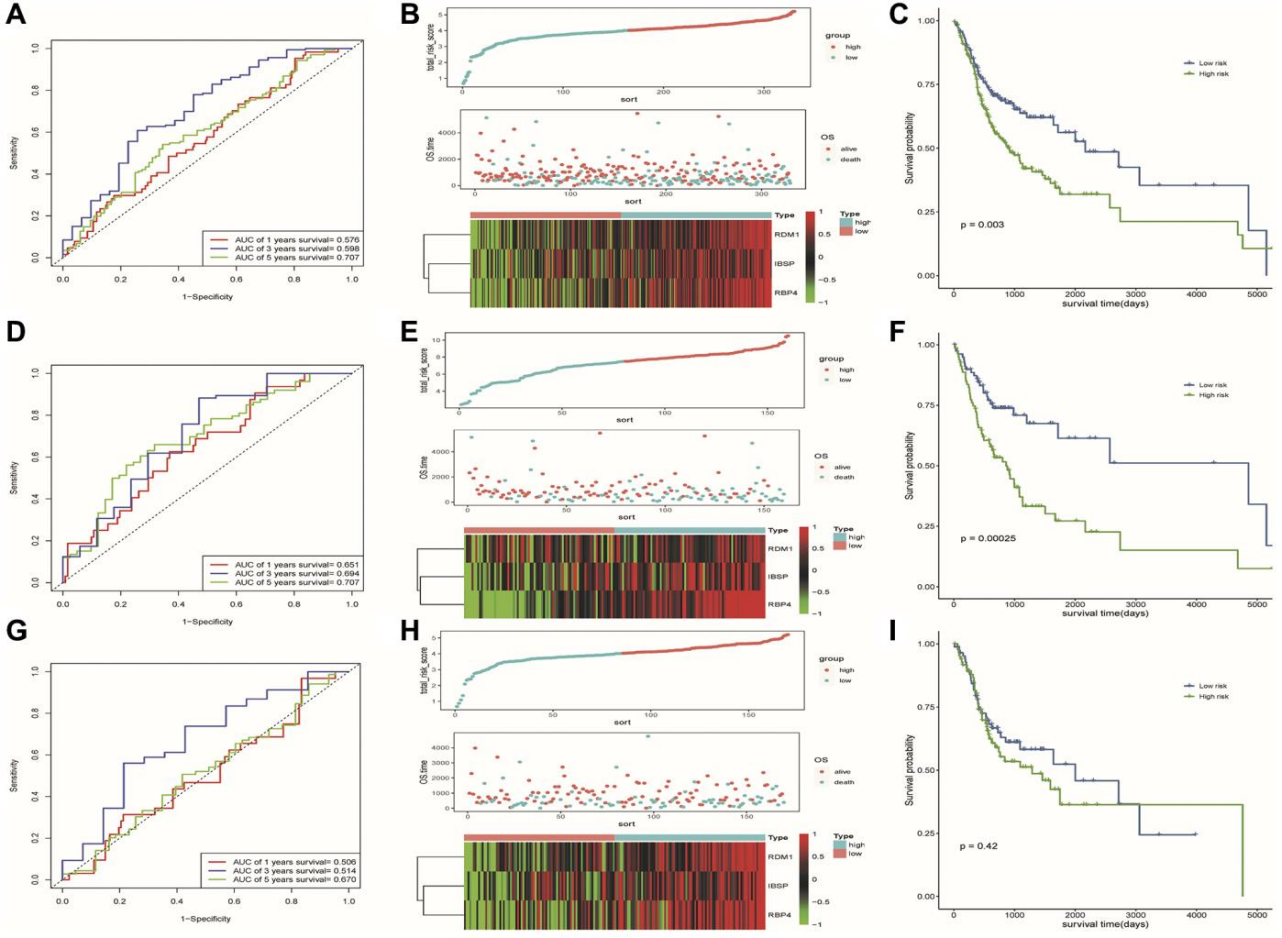
**Results of assessment of the risk model's ability to predict prognosis.** (**A**–**C**) ROC curve, riskplot and Kaplan-Meier curve of 3 DE-ERSs based on the all dataset. (**D**–**F**) ROC curve, riskplot and Kaplan-Meier curve of 3 DE-ERSs based on the training set; (**G**–**I**) ROC curve, riskplot and Kaplan-Meier curve of 3 DE-ERSs based on the testing set.

To evaluate the predictive ability of our risk model, we used the training set, test set, and all dataset simultaneously. It can be observed that as the risk score increased, the number of dead samples gradually increased. We observed that the Area Under Curve (AUC) in 1-, 3-, and 5-year Receiver Operating Characteristic Curve (ROC) for the risk model based on all dataset data were 0.576, 0.598 and 0.707, respectively (Figure 6A). In the test set, they are 0.506, 0.514, and 0.670 respectively, when they are identified as 0.651, 0.694, and 0.707 in the training set (Figure 6D, 6G). These results all indicated that our risk model has a good predictive value. Figure 6B, 6E, 6H showed the risk scores, survival status, and expression of three prognostic-related DE-ERSs in OSCC patients from all training and testing sets. The Kaplan-Meier survival curve also identified the excellent ability of risk model in distinguishing prognosis (Figure 6C, 6F). Unfortunately, we did not observe similar statistically significant results in training set (Figure 6I). To assess whether our model could be used as an independent predictor of prognosis in patients with OSCC, we performed univariate and multivariate Cox regression analyses based on risk scores and clinical factors. The results in Table 3 suggested that our risk model can be used as an independent prognostic indicator of prognosis in OSCC patients.

**Table 3:** Univariate/multivariate Cox regression analysis based clinical factors and risk score.

**Variables**	**Univariate analysis**		***P***	**Multivariate analysis**		***P***
	**HR**	**95% CI**		**HR**	**95% CI**	
TCGA training set						
Stage (I and II vs. III and IV)	1.38	0.84–2.28	0.21	1.29	0.77–2.15	0.33
Grade (G1 and G2 vs. G3 and G4)	1.26	0.75–2.10	0.38	1.26	0.75–2.11	0.38
Age (≤50 vs. >50)	1.27	0.66–2.45	0.47	1.36	0.70–2.65	0.36
Risk group (high/low)	2.38	1.48–3.84	**0.001**	2.35	1.45–3.82	**0.001**
TCGA testing set						
Stage (I and II vs. III and IV)	2.90	1.57–5.35	**0.001**	2.99	1.61–5.55	**0.00**
Grade (G1 and G2 vs. G3 and G4)	1.23	0.73–2.06	0.43	1.31	0.77–2.22	0.31
Age (≤50 vs. >50)	1.20	0.65–2.19	0.56	1.24	0.67–2.28	0.49
Risk group (high/low)	1.21	0.76–1.92	0.43	1.18	0.73–1.90	0.49
TCGA all dataset						
Stage (I and II vs. III and IV)	1.89	1.29–2.76	**0.001**	1.92	1.31–2.82	**0.001**
Grade (G1 and G2 vs. G3 and G4)	1.24	0.86–1.78	0.25	1.26	0.88–1.82	0.21
Age (≤50 vs. >50)	1.27	0.82–1.97	0.29	1.45	0.93–2.28	0.10
Risk group (high/low)	1.63	1.18–2.26	**0.001**	1.65	1.18–2.29	**0.001**

### Hierarchical analysis based on clinicopathological features and construction of nomogram for predicting prognosis

**Figure 7:**
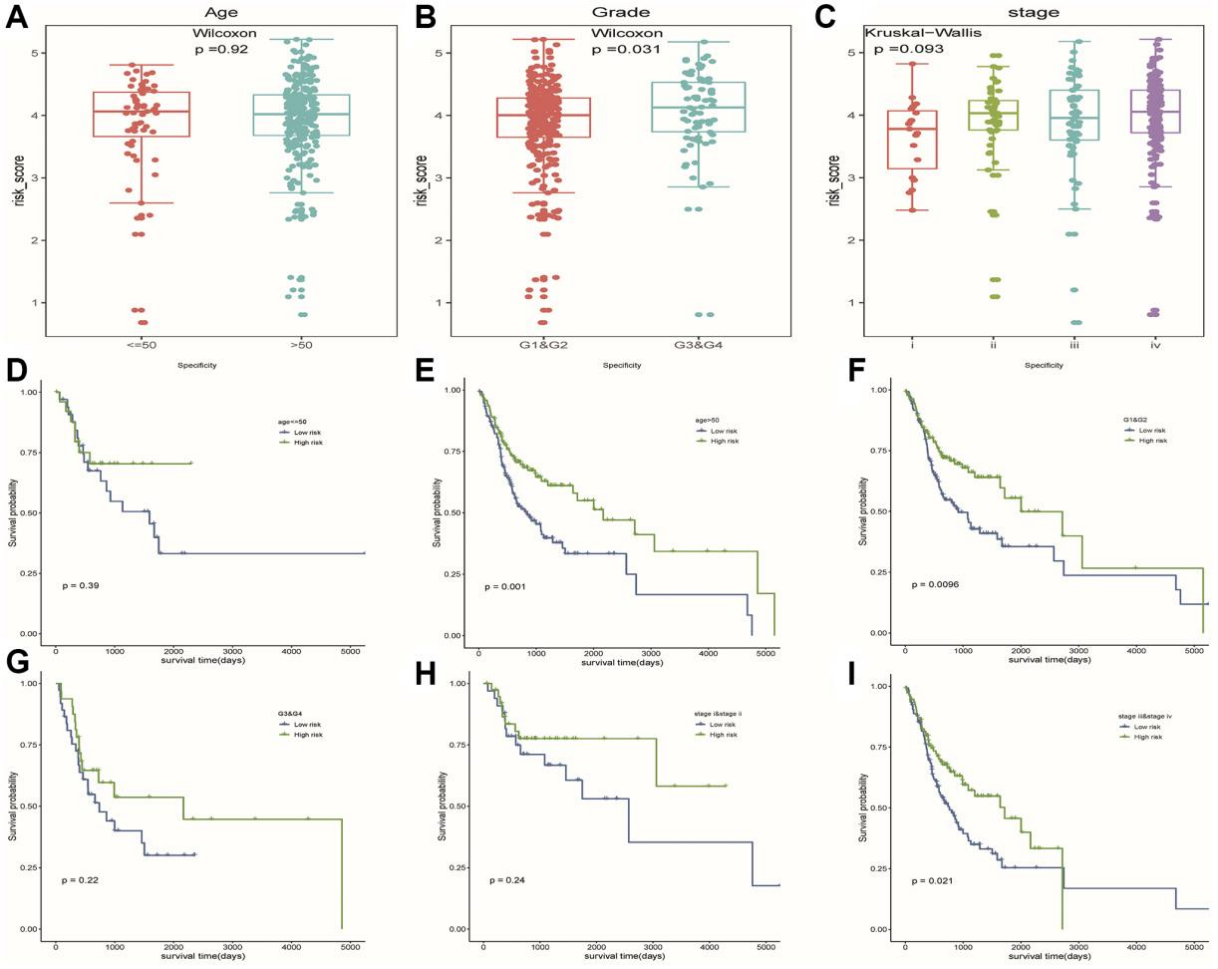
**The hierarchical analysis based on clinicopathological features.** (**A**–**C**) Differences of risk scores between patients with different clinicopathological characteristics (age, grade and stage), respectively. (**D**–**I**) Survival curves were used to assess the model’s ability to discriminate outcomes among subgroups of patients with different clinicopathological features. Blue and green represented low-risk and high-risk samples, respectively.

We observed higher risk scores in higher clinical grade samples (Figure 7B). In addition, no significant differences in risk scores were seen between/among subgroups of other clinical characteristics (Figure 7A, 7C). In a stratified analysis of clinicopathological features, we found that the risk model had a good ability to discriminate prognoses in patients with older than 50 years old (Figure 7E), tumor grades G1 and G2 (Figure 7F), or Clinical stage III and IV (Figure 7I). Unfortunately, we did not observe significant results in other subgroups of patients (Figure 7D, 7G, 7H).

**Figure 8:**
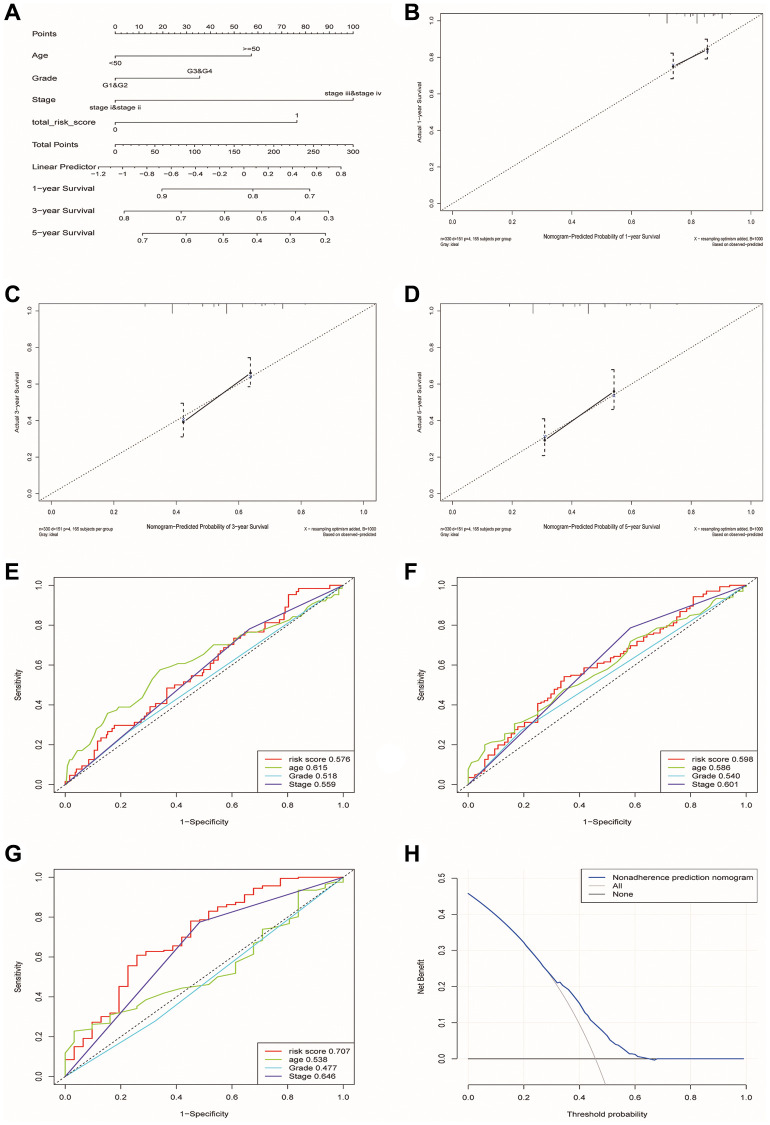
**Construction and verification of a nomogram for predicting the overall survival rate of patients with OSCC.** (**A**) Nomogram composed of age, grade, stage and risk group. (**B**–**D**) The calibration curve of Nomogram. The Y-axis represented the actual survival rate, while the X-axis represented the survival rate predicted by the Nomogram. (**E**–**G**) Multivariate ROC curves of 1, 3 and 5 years were used to predict prognosis based on nomogram. (**H**) The Decision Curve Analysis of the nomogram. The y-axis represents the net benefit. The blue and gray curves represented the net benefit of the model predictions and all interventions for all patients, respectively. In contrast, the horizontal line represented the net benefit of not accepting intervention for all patients. The intersection of the model curve and the All curve was the starting point, while the corner of the model curve and the None curve was the endpoint. Patients within this range could benefit.

Finally, we constructed a nomogram including four factors (age, stage, grade and risk group) that might affect prognosis (Figure 8A). The calibration plots showed that nomogram accurately estimated survival probabilities at 1, 3, and 5 years (Figure 8B–8D). The multivariate ROC curve also showed that the nomogram model had the highest AUC at 1, 3, and 5 years (0.576, 0.598, 0.707) compared with a single clinical factor (Figure 8E–8G). After evaluating the nomogram model’s benefit rate, the Decision Curve Analysis (DCA) was used to calculate the net benefit of model (Figure 8H). It could be seen from the figure that the combined model had a higher net benefit in the range of Pt of about 0.3~0.65 (Figure 8H).

### GEO database verification

**Figure 9:**
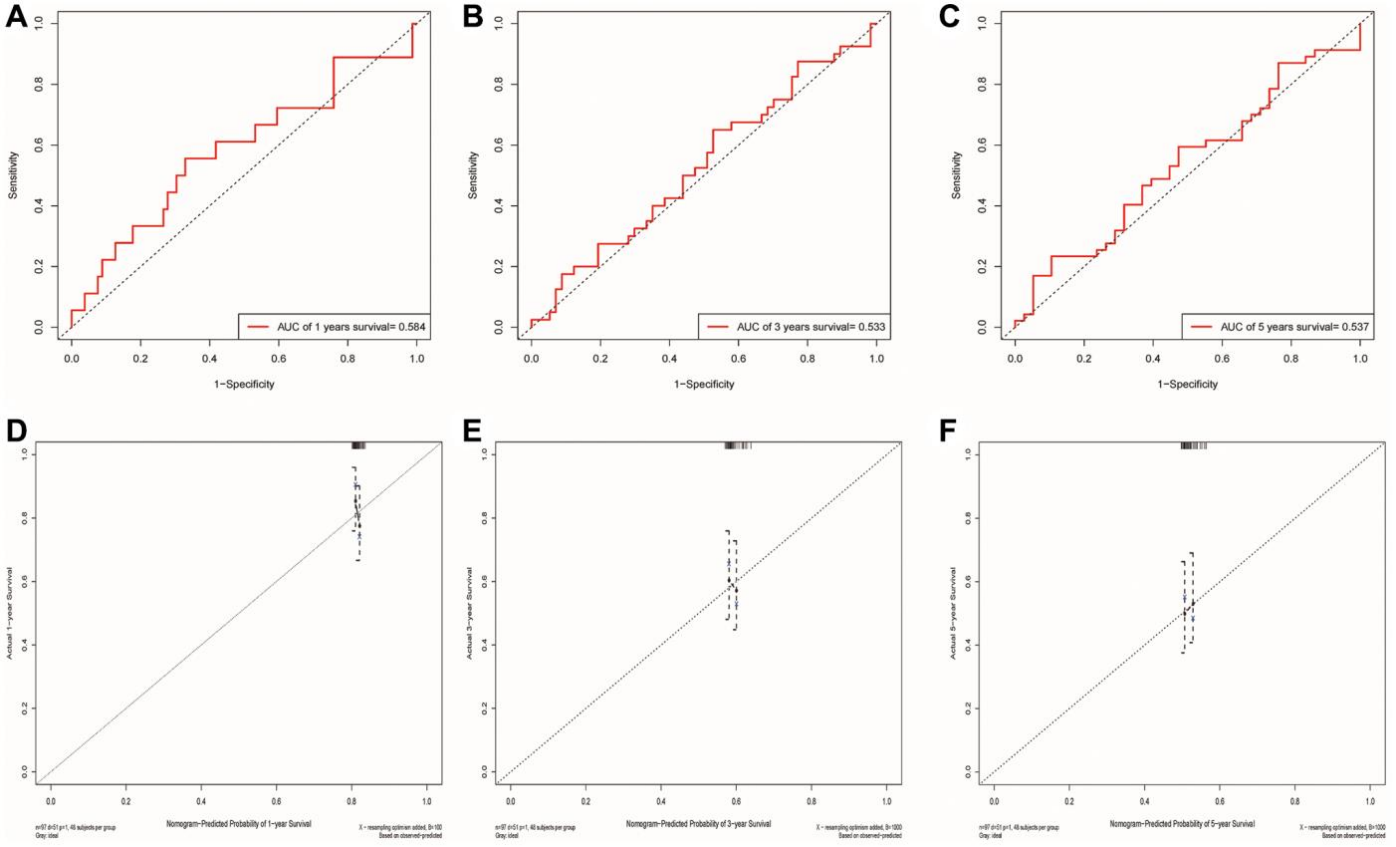
**Validation of the risk model and the ability of the nomogram to predict prognosis using the GSE41613 cohort.** (**A**–**C**) The ROC curve for predicting 1, 3 and 5-year survival rate based on the GSE41613 validation cohort, respectively. (**D**–**F**) Internal Calibration Curve of nomogram at 1-year, 3-year and 5-year based on the GSE41613 validation cohort, respectively.

We used the GSE41613 dataset containing clinical data for external validation of the model. The AUCs for 1-, 3-, and 5-year OS based on risk scores were 0.584, 0.533, and 0.537, respectively (Figure 9A–9C). The calibration curve of the nomogram also showed satisfactory agreement between the predicted and observed values of the 5-year OS probability in this cohort (Figure 9D–9F).

### Differences of tumor-infiltrating immune cells between different risk groups

**Figure 10:**
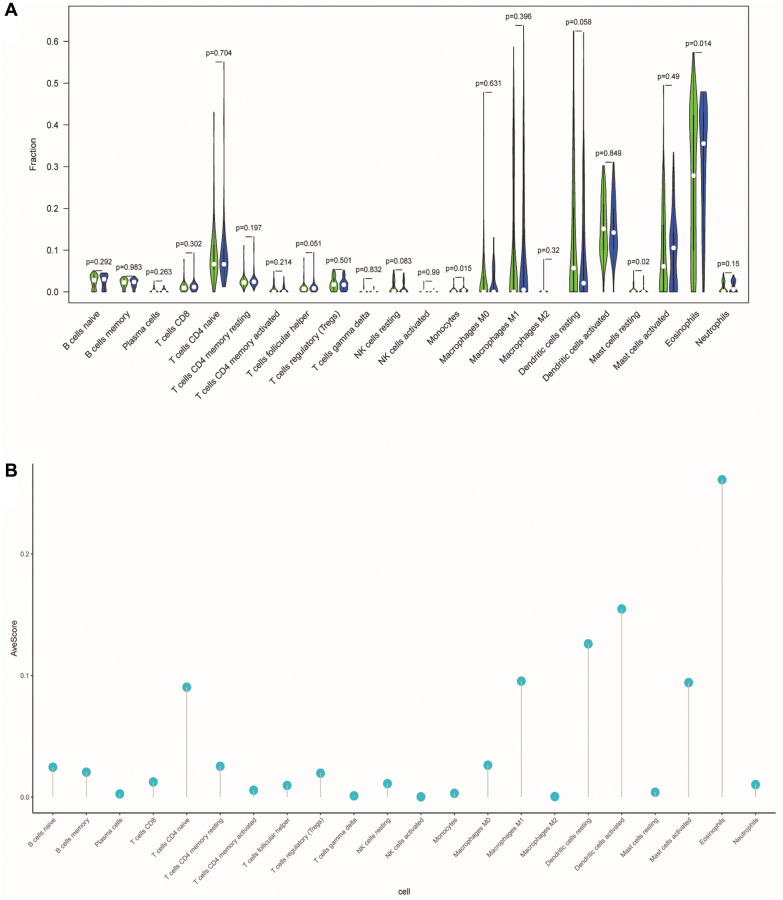
**Differences in tumor-infiltrating immune cells between different risk populations.** (**A**) The violin chart showed the difference in immune cells between the low-risk and high-risk group. Blue and green represented the low-risk group and the high-risk group, respectively. (**B**) The lollipop graph respectively showed the average relative content of the 22 immune cells in all TCGA samples.

To investigate the correlation between ERSs-based predictive risk model and the tumor immune microenvironment (TME), we visualized the differences in tumor-infiltrating immune cells between different risk groups (Figure 10A). There were significant differences in the contents of monocytes, resting mast cells and eosinophils between different risk groups (*p* = 0.015, *p* = 0.02 and *p* = 0.014), revealing the regulatory mechanism of TME in the occurrence and development of OSCC. Figure 10B showed the average score of each immune infiltrating cell.

### Correlation analysis of cell stemness

**Figure 11:**
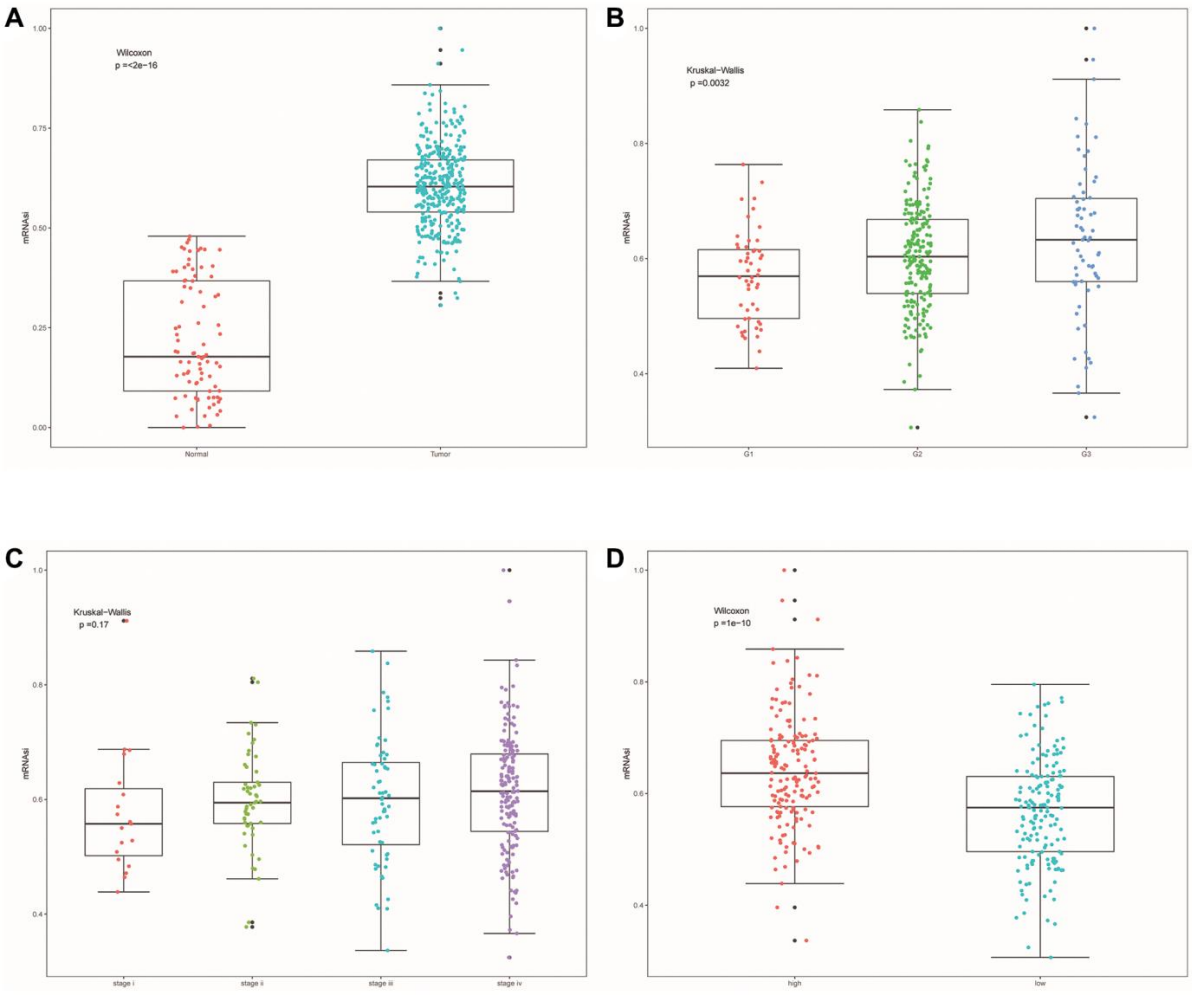
**Cell stemness correlation analysis.** (**A**) The differences of mRNAsi between OSCC samples and normal samples. (**B**) The differences of mRNAsi among different tumor grade subgroups. (**C**) The differences in mRNAsi among different clinical stage subgroups. (**D**) Differences in mRNAsi between other risk groups.

SsGSEA evaluated the dryness index (mRNAsi) of each OSCC sample of TCGA for us. Then we further explored the relationship between mRNAsi and classification/clinical characteristics/risk scores of patients (Figure 11A–11D). Unlike higher mRNAsi in OSCC patients (Figure 11A), we also observed significant differences in mRNAsi between different tumor grades and risk groups (Figure 11B, 11D). mRNAsi was observed to be higher in the G3 and high-risk groups.

### Drug sensitivity analysis

**Figure 12:**
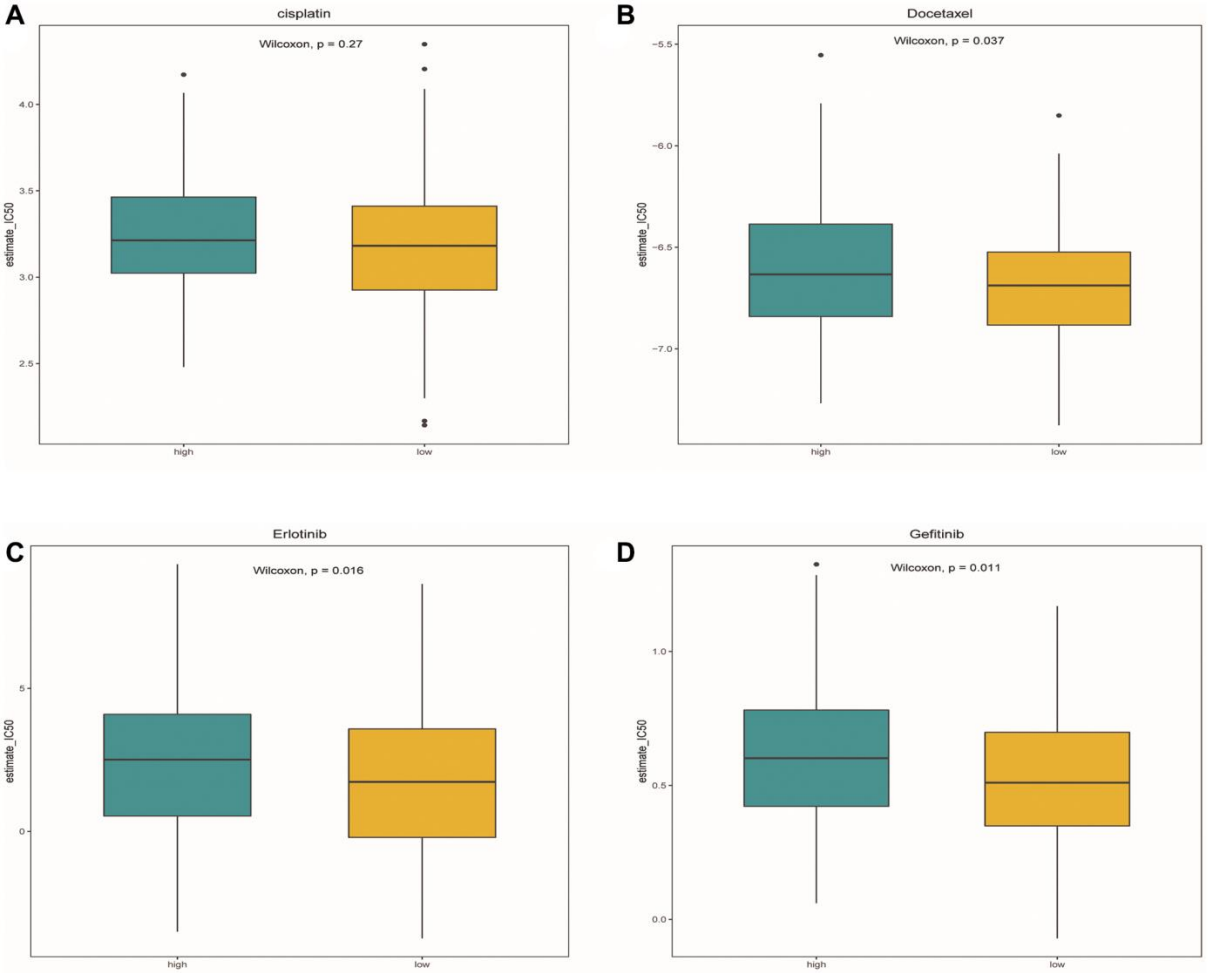
**Drug sensitivity analysis.** (**A**–**D**) Box plots showed the differences in IC50 of cisplatin, docetaxel, Gefitinib and erlotinib between the high-risk group and the low-risk group, respectively. Green represented the high-risk group, while yellow represented the low-risk group.

After comparing the IC50 differences between different risk groups, it was found that the high-risk group samples had higher IC50 of docetaxel, gefitinib and erlotinib (Figure 12A–12D). This means that low-risk patients are more sensitive to these three drugs. Perhaps in the future, we can predict the effect of these three drugs based on a patient’s risk score, leading to better treatment outcomes.

### Mutations associated with predictive model and prognosis

**Figure 13:**
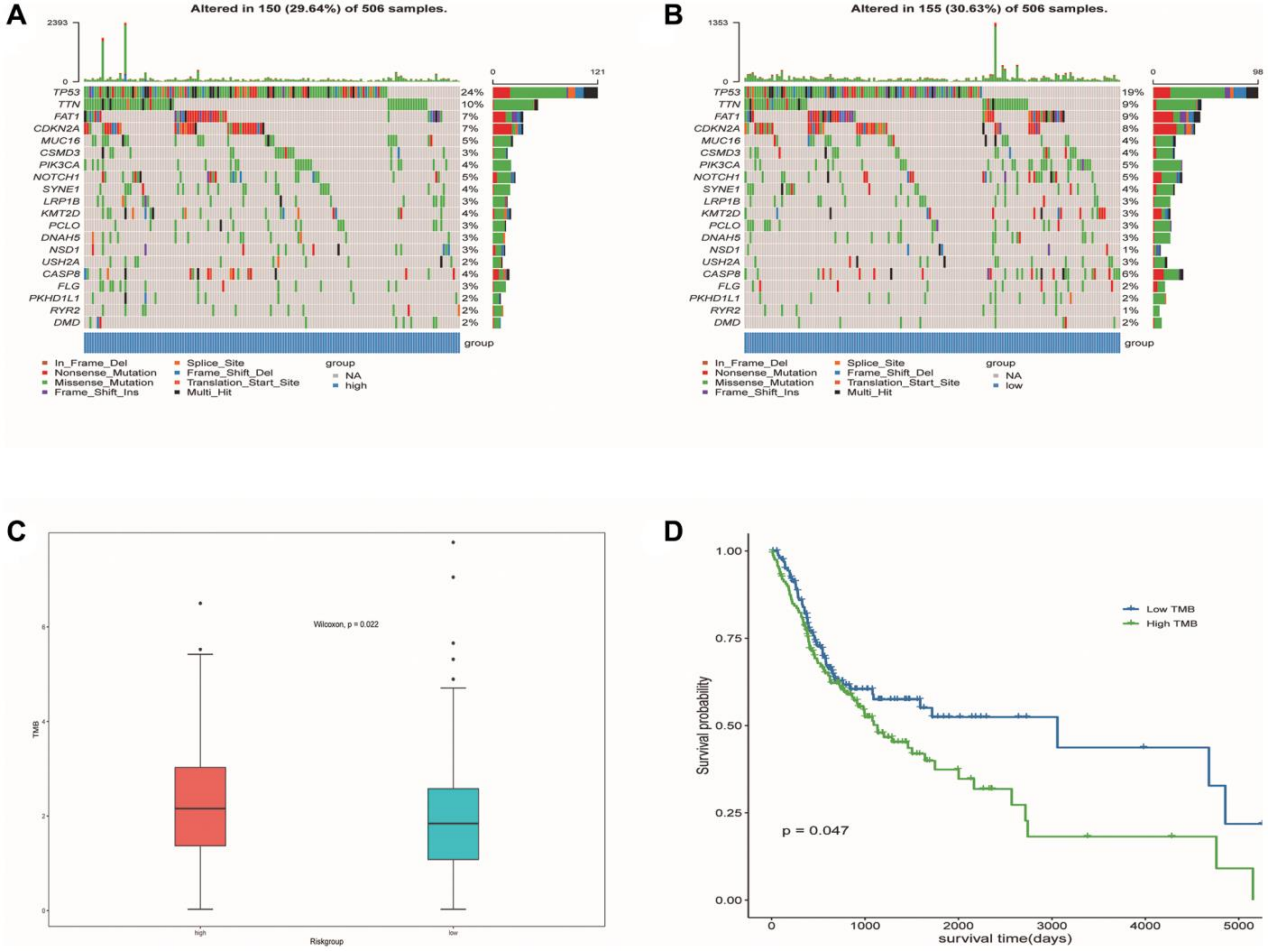
**Mutation profiled of low-risk and high-risk populations and TMB associated with model and OS.** (**A**) Mutation status of the high-risk population. (**B**) Mutation status of the low-risk population. (**C**) TMB associated with model based on DE-ERSs. (**D**) TMB associated with OS of OSCC samples.

We identified the top 20 most common gene mutations in both high- and low-risk groups. The corresponding statistical results were shown in Figure 13A, 13B. In addition, TMB in the high-risk group was significantly higher than in the low-risk group (Figure 13C). It can also be seen from the survival curve that there was a significant difference in the survival rate between the high TMB group and the low TMB group (Figure 13D). Patients with low TMB had a higher survival rate, suggesting that high TMB may have a negative influence on the prognosis of OSCC patients.

### Abnormal expression of three modeling genes in OSCC tissues and cells

**Figure 14:**
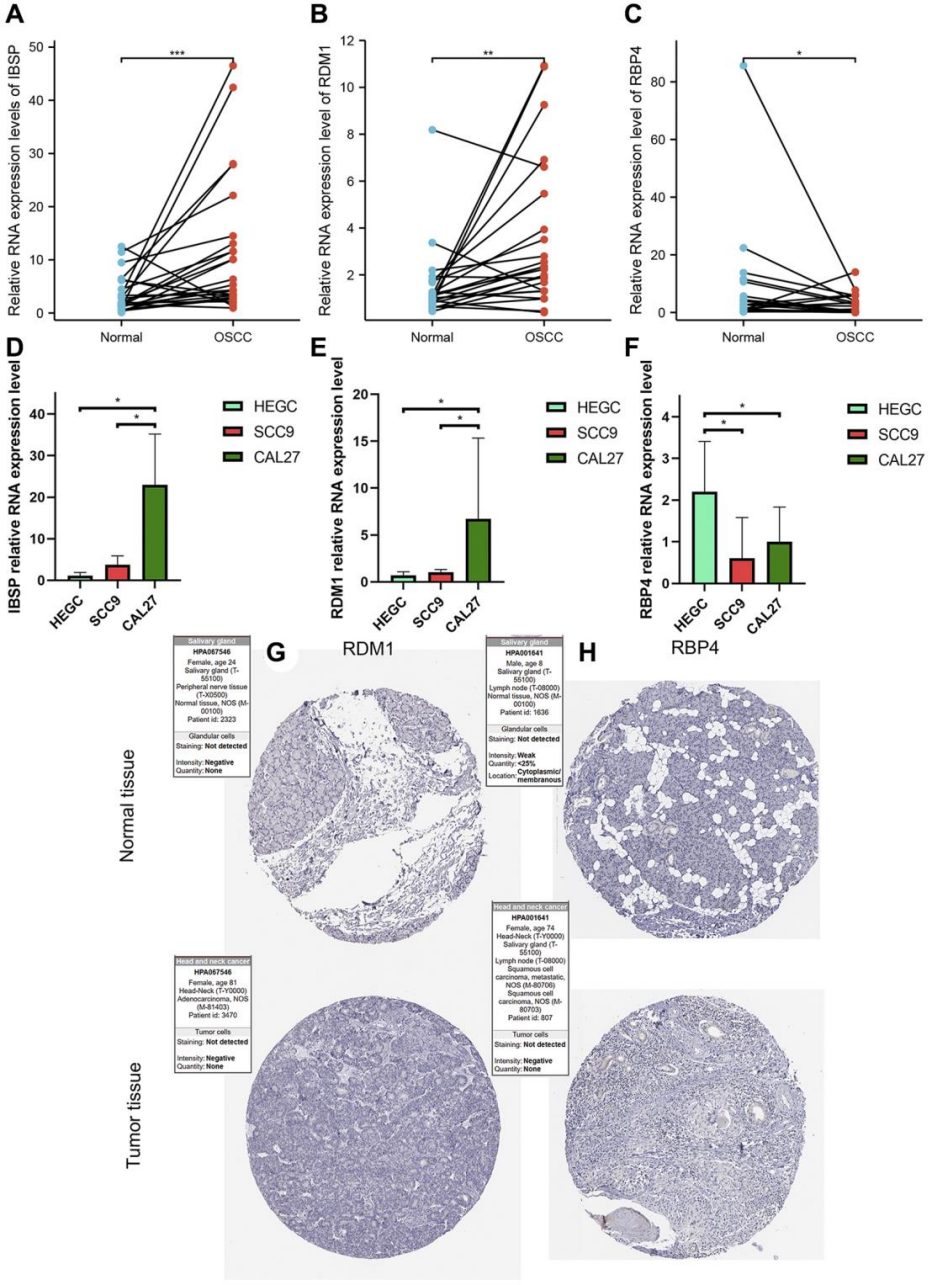
**Validation of abnormal expression of 3 modeled genes in OSCC.** (**A**, **B**) Higher relative mRNA expression levels of IBSP and RDM1 detected by QRT-PCR in OSCC tissues. (**C**) Lower relative mRNA expression levels of RBP4 detected by QRT-PCR in OSCC tissues. (**D**, **E**) Higher relative mRNA expression levels of IBSP and RDM1 detected by QRT-PCR in cal27 and scc9. (**F**) Lower relative mRNA expression levels of RBP4 detected by QRT-PCR in cal27 and scc9. (**G**, **H**) IHC images reflecting the expression of RDM1 and RBP4 proteins in normal oral tissues and head and neck squamous cell carcinoma tissues. ^*^p < 0.05; ^**^p < 0.01; ^***^p < 0.001.

From the results of paired *t*-test, we observed that the RNA levels of IBSP and RDM1 were significantly higher in OSCC tissues (Figure 14A, 14B). Furthermore, relative RNA levels of RBP4 were observed to be lower in OSCC tissues (Figure 14C). In addition, further cell experiments also confirmed significantly higher IBSP, higher RDM1 and lower RBP4 relative RNA levels in scc9 and cal27 (Figure 14D–14F). Unfortunately, we did not detect differential protein expression of RDM1 and RBP4 between normal oral and tumor tissues (Figure 14G, 14H).

## DISCUSSION

As the most common oral cancer, OSCC has a poor prognosis and high mortality [25]. The molecular pathogenesis of OSCC is complex, and there is a lack of accurate biomarkers to predict the prognosis of patients. At present, some studies have analyzed the link between ER stress and cancer. Previous studies have found that ER stress can promote potential pancreatic tumor metastasis [26]. Wu et al. found that ER stress can drive tumorigenesis and progression of HCC [18]. ER stress also plays a dynamic reprogramming role in promoting tumor growth, invasion, therapeutic resistance, and infiltration of immune cells in brain tumors [27]. After we checked the database, we found that the use of ERSs to predict the prognosis of OSCC patients is very rare, so it is necessary to identify prognostic markers related to ER stress in OSCC. In this study, the DE-ERSs generated by the string database was used to construct a PPI network, and the MCC algorithm was used as the hub gene.

To clarify the biological processes involved in DE-ERGs, we ran GO and KEGG enrichment analysis. After running lasso regression and multiple Cox regression analysis, a prognostic risk model was constructed using the three prognostic-related DE-ERSs. Prognostic evaluations based on the training set, test set, and all datasets all confirmed the good prognostic prediction performance of the risk model. In addition, risk score was identified as an independent prognostic factor. The nomogram composed of multiple factors provided an accurate quantitative tool for prognosis prediction.

Through enrichment analysis of the GSEA pathway, we were surprised to find that DE-ERSs were significantly enriched in signaling through GPCR, GPCR ligand binding, nuclear receptor signaling, and other pathways. Our further study and analysis found that the activation of GPCRs expressed in various cells can stimulate ER stress [28]. Furthermore, OGR1 was identified as an example of GPCR protein expression in gut-related inflammatory diseases that regulates ER stress through the IRE1 α-JNK signaling pathway, a body of evidence that forms a strong understanding between GPCRs and ER stress [29]. Epithelial-Mesenchymal Transition (EMT) is a process in which epithelial cells lose apical-basal polarity and strong cell contacts and gain spindle shape and greater motility [30]. We found that both GPCR and ER stress plays a role in EMT [31, 32]. This process is critical in physiological phenomena such as embryogenesis and wound healing and pathological events [33, 34]. Chemotactic migration is a crucial aspect of EMT and cancer progression [35]. Undoubtedly, this will provide a guiding direction for the corresponding research in the future. On the other hand, the coordinating role of ER stress in EMT initiation has been well established [36–38]. Hypoxia is a driver that promotes EMT transcription factors and activates ER stress markers in rat lungs and alveolar epithelial cells [38]. Hypoxia and intracellular calcium are involved in the EMT induction of AECs, mainly by activating ER stress and the hypoxia-inducible factor signaling pathway [39].

Interestingly, we found the significant differences in the content of monocytes, resting mast cells, and eosinophils between high- and low-risk groups. After intensive research, we discovered that ER stress affects the response of human monocytes to their ability to differentiate into macrophages [40]. The ability of monocytes to differentiate into macrophages is affected by inhibition, monocytes are less responsive to endotoxin. Monocytes are progenitor cells that differentiate into mature resident macrophages in various human tissues and have emerged as important immune regulators controlling adaptive immune responses [41]. An obstacle to nuclear cells is becoming macrophages [42]. We also found that chronic insulin exposure induces ER stress and lipid volume accumulation in mast cells, disrupting the secretory degranulation response of mast cells, thereby reducing mast cells in high-risk populations [43]. The RDM1 gene is identified through database searches for proteins similar to RAD52. Like RAD52, RDM1 participates in DNA double strand break repair and homologous recombination [44, 45]. Increased RDM1 mRNA expression was closely associated with decreased overall survival and progression-free survival [44, 46]. In addition, the increased expression of RDM1 mRNA was closely associated with the infiltration levels of macrophages, CD8-T cells, and B cells. RDM1 can regulate the expression of p53 [46–49]. P53 plays an essential role in regulating cell growth and apoptosis [47]. The negative regulation of p53 by IBSP is a binding molecule that plays a crucial role in protein migration and cell surface adhesion [50]. It promotes the formation of transfer factor precursors by stimulating molecular signals to form adherent plaques [51–53]. According to our research, IBSP is also associated with EMT. In addition, studies have also shown that IBSP can enhance the proliferation and tumor metastasis of ESCC cells [54]. In our study, bioinformatics analysis, tissue and cell experiments have fully verified the significantly higher expression of IBSP, RDM1 in OSCC. The significantly lower expression of RBP4 in OSCC was also well confirmed. These results fully showed that these three genes may play an important biological role in OSCC. In addition, the evidence also supported the contribution of these three modeling genes to the stability of the model.

This study found that TMB in the high-risk group was significantly higher than that in the low-risk group through differential analysis. Patients with low TMB had a higher survival probability, indicating that high TMB may play a negative role in the prognosis of OSCC patients. It is well known that TMB has been regarded as an effective predictor of the efficacy of immunotherapy, like PDL1 [55]. This also reveals that high-risk patients with high TMB may benefit more from immunotherapy. In the drug sensitivity analysis, we observed a higher sensitivity to docetaxel in the low- risk group. Therefore, we speculate that, in low-risk patients, docetaxel may induce endoplasmic reticulum-mediated activation of ER stress signaling-related proteins GRP78, ATF6, IRE1α, and PERK/eIF2α, resulting in docetaxel-induced Apoptosis [56].

Our study constructed a prognostic risk model with good predictive performance in OSCC. It was also assessed to have outstanding value in guiding treatment. We also found that it may participate in the progression of OSCC through immune infiltrating cells, cell stemness and TMB. But our study was statistically significant for OS to predict prognosis in OSCC. However, our study also has some limitations: 1. Many conclusions are indeed difficult to carry out basic experiments to verify their reliability; 2. Limited data sets limit further verification of model performance. 3. The performance of the prognostic risk model we established needs to be tested by clinical samples in the future.

## MATERIALS AND METHODS

### Data sources

**Figure 15:**
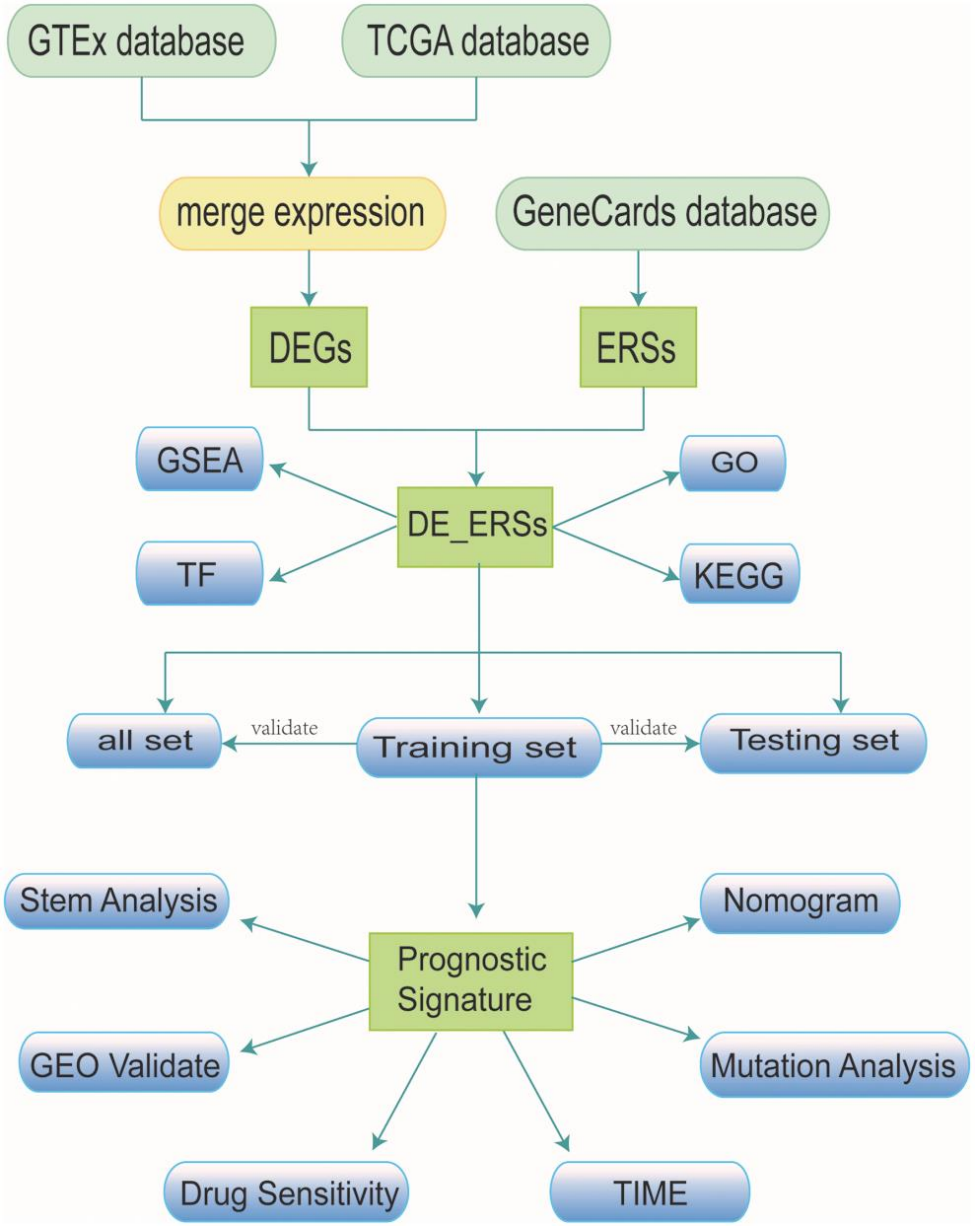
Research workflow.

The process of our study was presented as a flow chart in Figure 15. To carry out the next analysis, we first downloaded the corresponding data of head and neck squamous cell carcinoma (HNSC) from The Cancer Genome Atlas (http://cancergenome.nih.gov/, TCGA) database. Next, we centrally extracted 331 cases whose main sites were in the oral cavity (tongue, lips, cheek, palate, gums, floor of mouth, etc.) with somatic mutations, RNA sequencing and corresponding clinical data. In addition, we obtained transcriptome data of 55 normal minor salivary gland tissue samples in the Genotype Tissue Expression (http://gtexportal.org/, GTEx) database [57]. To lay the foundation for follow-up research, we carried out log2 transformation of the RNA sequencing value of each gene. [58]. From the gene card database (http://genecards.org/, GeneCards) that automatically integrated gene-centric data from ~150 web sources, including genomic, transcriptomic, proteomic, genetic, clinical and functional information, we obtained 5885 endoplasmic reticulum stress-related genes (ERSs) with correlation coefficient >1 [59].

### Identification of differentially expressed genes

After integrating the data of TCGA and GETx, we used the “limma” R package to analyze the difference in gene expression value between OSCC and normal tissues. The genes filtered according to the threshold |log2(FC)| >1 and *P*-value < 0.05 were defined as differentially expressed genes (DEGs) [60]. While the genes with log2(FC) >1 were identified as DEGs with up-regulated expression, genes with log2(FC) < −1 were identified as DEGs with down-regulated expression.

### Construction of protein-protein interaction network

The DEGs of OSCC and 5885 ERSs were intersected to obtain the common differentially expressed genes of endoplasmic reticulum stress (DE-ERSs). The STRING was known as a database for searching known proteins and predicting protein-protein interactions [61]. We selected De-ERSs with a combined score >400 to construct a protein-protein interaction network (PPI), which was visualized by Cytoscape [62]. According to previous studies, the Maximal Clique Centrality (MCC) algorithm has been identified as the most effective method for finding hub genes in the co-expression network [63]. After calculating the MCC of each node through the CytoHubba plug-in in Cytoscape, the top 20 genes with MCC values were regarded as hub genes and the corresponding network diagram was visualized through the NetworkAnalyzer online tool [64].

### Functional enrichment of DE-ERSs

KEGG was known as a database that was widely used to store information about genomes, biological pathways, diseases and drugs, etc. GO functional annotation analysis was widely used to enrich gene functions on a large scale, including biological process (BP), molecular function (MF) and cellular component (CC). Disease Ontology (DO), a kind of enrichment analysis of diseases based on genes, played an important role in understanding the pathogenesis of complex diseases based on similar relationships between diseases, early prevention and diagnosis of major diseases, new drug development, and drug safety assessment [65]. In our study, we not only used the R package “cluster profiler” to perform GO functional annotation analysis and KEGG pathway enrichment analysis on De-ERS, but also applied the “DOSE” package to enrich diseases [66, 67].

### Enrichment analysis of DE-ERSs

To judge the contribution of gene sets to phenotypes, we used Gene Set Enrichment Analysis (GSEA), which can be used to assess gene distribution trends in predefined gene sets ranked by phenotypic relevance in the gene table [68]. We applied the “c2.cp.v7.4. symbols” gene set obtained in the MSigDB database (http://gsea-msigdb.org/gsea/msigdb) to the DE-ERSs-based GSEA analysis. In this process, we used the R package “cluster profile” and got the biological process map related to the enrichment pathway results from the REACTOME database (http://reactome.org/) [69].

### Univariate Cox regression analysis for prognosis-related DE-ERSs

Univariate Cox regression analysis was applied to assess the association between expression values of DEGs and Overall Survival (OS) in 330 samples with complete survival data. And based on the screening condition with a threshold of *p* < 0.05, DEGs with a significant impact on prognosis were selected. We further extracted the common genes of these De-ERSs and ERSs and defined them as prognosis-related DE-ERSs [69].

### Construction and verification of prognostic risk model

After screening out patients with incomplete clinical data, we finally obtained 330 samples with complete OS information and divided these 330 samples into a training set (*n* = 160), test set (*n* = 170). In the following analysis, the data of three sets will be used to verify this model. 10 DE-ERSs obtained by running univariate cox regression on the training set data were used to run lasso regression (lasso) and multivariate Cox regression analysis for construction of prognostic risk model. As a shrinkage estimation method, lasso aims to minimize the residual sum of squares under the constraint that the sum of the absolute values of the regression coefficients is less than a constant, so as to generate some regression coefficients strictly equal to 0 to construct model. According to the calculation method: risk score = exp gene 1 × β gene 1 + exp gene 2 × β gene 2 + exp gene 3 × β gene 3 + exp gene n × β gene n (where exp gene n represents gene expression level, β gene n represents the regression coefficient calculated by multivariate COX regression), we calculated the risk score of all samples. Patients were divided into high- and low-risk groups for subsequent validation based on the median risk score. The distribution of survival status for each patient was shown separately on a dot plot according to the risk score ranking for each sample. Kaplan-Meier survival curves and time-dependent ROC curves were used to assess the accuracy of predicting prognosis with risk scores. In addition, univariate and multivariate Cox were used to identify independent prognostic factors.

### Hierarchical analysis based on clinicopathological features and construction of nomogram for predicting prognosis

To assess the relationship between clinicopathological features and risk scores, we compared the differences of risk scores among different subgroups of clinicopathological features, such as age, grade and stage. In addition, we performed a stratified analysis of clinicopathological characteristics to assess the ability of the prognostic risk model to predict the prognosis in different subgroups. We used potential prognostic factors to establish a nomogram to predict the 1-, 3- and 5-year survival rates of patients with OSCC. By comparing the predicted value of the nomogram with the observed actual survival rate, a calibration curve was generated to evaluate the performance of the nomogram. Furthermore, the multi-factors ROC curve was not only used to verify the accuracy of the nomogram, but also the optimality in predicting 1-, 3-, and 5-year survival rate. The DCA curve is a simple method to evaluate clinical predictive models, diagnostic tests and molecular markers. After confirming the accuracy, we assessed and visualized the net benefit of the nomogram model through the DCA curve.

### Verification based on GEO database

The dataset GSE41613 from the Gene Expression Omnibus (http://ncbi.nlm.nih.gov/geo/, GEO), containing data from 97 patients with OSCC, was used as the validation cohort. ROC curves were again used to assess the ability of the prognostic risk model in predicting prognosis. Internal calibration curves were further used to evaluate the accuracy of the nomogram in prognostic prediction.

### Correlation between model and immune infiltrating cells

The RNA sequencing data of the OSCC dataset was used to estimate the proportion of immune infiltrating cells in each sample based on the CIBERSORT algorithm, which was widely known for deconvoluting the expression matrix of immune infiltrating cell subtypes based on the principle of linear support vector regression. Subsequently, we compared the differences in the proportion of each type of immune infiltrating cells between different risk groups.

### Correlation between model and cell stemness

The Single-Sample Gene Set Enrichment Analysis (ssGSEA) algorithm is an algorithm that associates a specific cell type with a set of signature genes. As a special GSEA, ssGSEA is mainly used for a single sample that cannot be a GSEA [70]. As an extension of the GSEA principle, it calculates the rank value of each gene based on the expression profile file for subsequent statistical analysis [70]. Unlike GESA, ssGSEA does not prepare expression profile files in gct format, but each sample’s score is under the corresponding background gene set [70]. Based on ssGSEA, the mRNA expression-based stemness index (mRNAsi) of OSCC samples was evaluated using tumor stem cell gene collection. In addition, mRNAsi was mapped to the 0–1 range by a linear transformation that subtracted the minimum value and divided it by the maximum value.

### Drug sensitivity analysis

In the study, to evaluate whether our model can be used as a marker for predicting clinical efficacy, we used the Genomics Database of Cancer Drug Sensitivity (http://cancerrxgene.org/, GDSC) to estimate the sensitivity of four chemotherapeutic drugs (cisplatin, docetaxel, gefitinib, and erlotinib) routinely used to treat OSCC in each sample of OSCC [71]. After setting all parameters to default values, use the R package “pRRophetic” to run ridge regression. A prediction model was designed based on the GDSC cell line dataset using ridge regression, and its satisfactory prediction accuracy was evaluated using 10-fold cross-validation. Based on the predictive models for these 4 drugs, the half maximal inhibitory concentration (IC50) was estimated for each OSCC sample in the TCGA dataset.

### Mutation analysis

An analysis was performed using somatic mutation data from 330 MAF-formatted OSCC samples in the TCGA database to assess the relationship between gene mutations and prognostic risk model. The R package “maptools” is an efficient way to aggregate, analyze, annotate and visualize MAF files from TCGA sources or any in-house study. We use R-packet “maptools” to count and visualize the top 20 genes’ mutations in high-risk and low-risk populations. Tumor mutational burden (TMB), defined as the total number of somatic mutations with substitutions, insertions/deletions per Mb in the exon coding region of the genome, was calculated for differential analysis. To explore the effect of TMB on survival rate, we first divided the samples into low and high groups by median TMB. The difference in survival rate between the two groups was then compared by Kaplan-Meier analysis.

### Abnormal expression of three modeling genes in OSCC was verified by *in vivo* and *in vitro* experiments

To further verify the differences in the transcription levels of these three genes between OSCC and normal oral tissue, we further detected the relative mRNA expression levels of these genes by quantitative real-time PCR (QRT-PCR) experiment. The Ethics Committee of the Affiliated Stomatological Hospital of Nanchang University (2021-08-015) approved this study and patients consented to specimen collection. 24 matched pairs of OSCC and adjacent paracancerous oral tissues came from the subjects who underwent surgery. The primer sequences of all genes were listed in Table 4.

**Table 4:** All primer sequences used in QRT-PCR experiment.

**Gene**	**Forward primer**	**Reverse primer**
**β-Actin**	TGGCACCCAGCACAATGAA	CTAAGTCATAGTCCGCCTAGAAGCA
**IBSP**	CCCCACCTTTTGGGAAAACCA	TCCCCGTTCTCACTTTCATAGAT
**RDM1**	TCCAGTCAAGGTTCGTCTTGG	TGGCATTTGGAACTGTTCAGG
**RBP4**	AGGAGAACTTCGACAAGGCTC	GAGAACTCCGCGACGATGTT

Total RNA extracted from tissues using the TransZol Up Plus RNA Kit (TRANS, Beijing, China) was reverse transcribed into cDNA using EasyScript First-Strand cDNA Synthesis SuperMix (TRANS, Beijing, China). QRT-PCR was performed using the Roche Light Cycler 96 Real-time Fluorescent Quantitative PCR System (Roche Applied Science, Mannheim, Germany) and Taq Pro Universal SYBR qPCR Master Mix (Vazyme, Nanjing, China). After normalizing all measured values to relative expression levels of β-actin using the 2^−ΔΔCt^ method, we compared differences in the expression levels of 3 modeling gene between paired tissues using paired *t*-tests.

Human tongue squamous cell lines cal27 and scc9 were purchased from Shanghai Anwei Biotechnology Co., Ltd. (Shanghai, China). Normal oral gingival epithelial cell line HEGC were purchased from Shanghai Baiye Biotechnology Center (Shanghai, China). HGEC and cal27 were cultured in DMEM (Gibco, Cat#C11995500BT), while scc9 was cultured in DMEM/F12 (Gibco, Cat#C11330500BT). All media contain 10% Fetal Bovine Serum (Excell, Cat#FSP500) and 1% Penicillin-Streptomycin Liquid (Solarbio, Cat#P1400). All cells were cultured at 37°C in 5% CO2’s humidified incubator. To further verify the abnormal expression of the three modeling genes in OSCC cells, we again used QRT-PCR to detect the relative RNA expression of the three modeling genes in scc9, cal27 and HEGC.

### Immunohistochemical (IHC) staining verifies the abnormal expression of modeling genes in tumor

We downloaded IHC images reflecting the expression of RDM1 and RBP4 proteins in normal oral tissues and head and neck squamous cell carcinoma tissues in the Human Protein Atlas (http://proteinatlas.org/, HPA) database. Next, we compared the differences between the protein expression of these two genes in normal oral tissue and tumor tissue.

### Statistical analysis

All data calculations and statistical analyses were performed in R software (version 3.6.2). Depending on the distribution characteristics, we used Student’s *t*-test or Mann-Whitney test to compare the difference between continuous variables. The chi-square test or Fisher’s exact test was used to compare the difference between categorical variables. All statistical *P*-value are two-sided, and the results with *P* < 0.05 are regarded as statistically significant.

## Supplementary Materials

Supplementary Figure 1
