# Supplementary material for: Identification of an endoplasmic reticulum stress-related prognostic risk model with excellent prognostic and clinical value in oral squamous cell carcinoma
Source: Aging (Albany NY). 2023 Aug 25;15(19):10010–30. doi: 10.18632/aging.204983 (PMC10599730; doi:10.18632/aging.204983)
Supplement: Supplementary Figure 1 [file aging-15-204983-s001.pdf]

SUPPLEMENTARY FIGURE

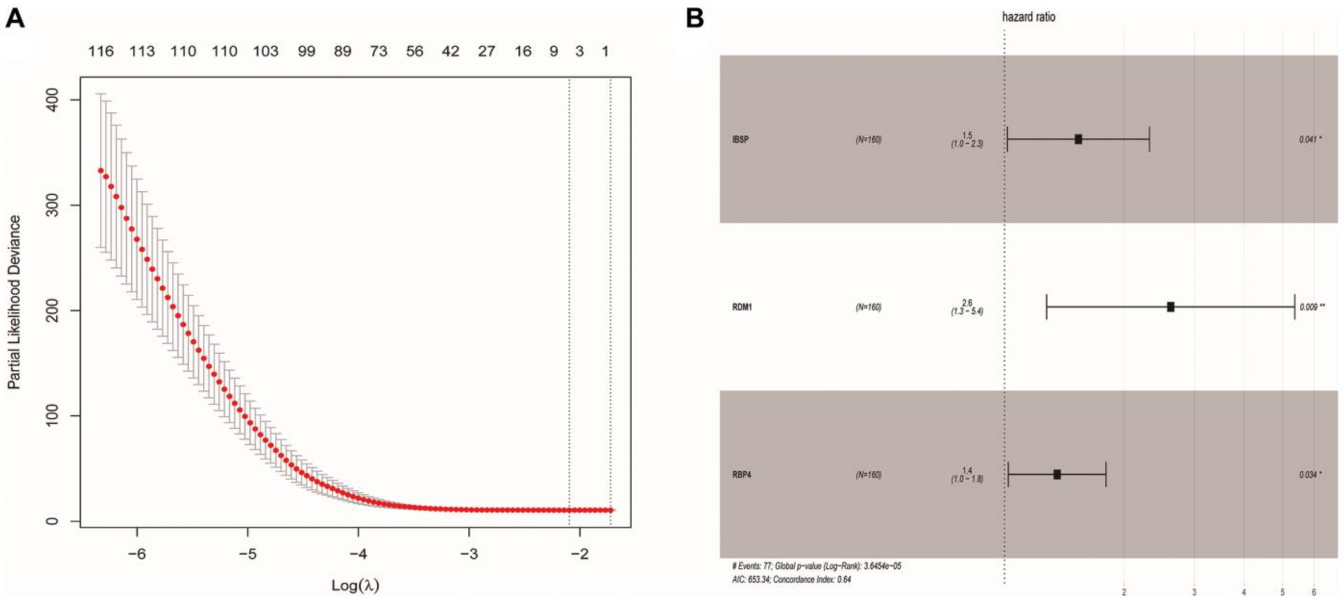

Supplementary Figure 1. The process of screening modeling genes by Lasso and univariate Cox regression. (**A**) The vertical dashed line represented the lambda value with the minimum error and the maximum lambda value. (**B**) The forest map showed the results of univariate Cox regression analysis of three risk model genes. The left and right sides of the vertical dotted line represent protective genes and risk genes, respectively.
